# Research of 2D-COS with metabolomics modifications through deep learning for traceability of wine

**DOI:** 10.1038/s41598-024-63280-9

**Published:** 2024-06-01

**Authors:** Zhuo-Kang Wang, Na Ta, Hai-Cheng Wei, Jin-Hang Wang, Jing Zhao, Min Li

**Affiliations:** 1 School of Electrical and Information Engineering, North Minzu University, No. 204 North Wenchang Street, Yinchuan, 750021 Ningxia China; 2 School of Medical Technology, North Minzu University, No. 204 North Wenchang Street, Yinchuan, 750021 Ningxia China; 3 School of Information Engineering, Ningxia University, Yinchuan, 750021 China; 4 College of Biological Science and Engineering, North Minzu University, Yinchuan, 750021 Ningxia China

**Keywords:** NIR, Traceability of wine origin, 2D-COS, Convolutional neural network, Metabolomics, Chemistry, Engineering

## Abstract

To tackle the difficulty of extracting features from one-dimensional spectral signals using traditional spectral analysis, a metabolomics analysis method is proposed to locate two-dimensional correlated spectral feature bands and combine it with deep learning classification for wine origin traceability. Metabolomics analysis was performed on 180 wine samples from 6 different wine regions using UPLC-Q-TOF-MS. Indole, Sulfacetamide, and caffeine were selected as the main differential components. By analyzing the molecular structure of these components and referring to the main functional groups on the infrared spectrum, characteristic band regions with wavelengths in the range of 1000–1400 nm and 1500–1800 nm were selected. Draw two-dimensional correlation spectra (2D-COS) separately, generate synchronous correlation spectra and asynchronous correlation spectra, establish convolutional neural network (CNN) classification models, and achieve the purpose of wine origin traceability. The experimental results demonstrate that combining two segments of two-dimensional characteristic spectra determined by metabolomics screening with convolutional neural networks yields optimal classification results. This validates the effectiveness of using metabolomics screening to determine spectral feature regions in tracing wine origin. This approach effectively removes irrelevant variables while retaining crucial chemical information, enhancing spectral resolution. This integrated approach strengthens the classification model's understanding of samples, significantly increasing accuracy.

## Introduction

The geographical origin of wine has a significant impact on its sensory characteristics, chemical composition, and commercial value^1^. With the improvement of people's living standards and the surge in global wine sales, inferior wine with forged geographical labels has seriously threatened consumer health and industry development^2^, and wine origin traceability technology has gradually attracted public attention.

At present, the provenance traceability technology of wine mainly depends on stable nuclide technology, gas chromatography–mass spectrometry and high-performance liquid chromatography. For example, Sudol et al. used two-dimensional gas chromatography-time-of-flight mass spectrometry to analyze the volatile components of five white "Grillo" wines from Sicily, found the differential characteristic components that can characterize geographical labels, and carried out a traceability study on them^3^. Zhang et al. analyzed the Anthocyanidin derivatives of 234 red wines in different years by liquid chromatography-mass spectrometry, and classified the wines in different years and aging stages^4^. Wu et al. used Stable nuclide technology, element analysis and Chemometrics methods to screen six elements, such as Mg, Mn and Na, as important variables, to trace the origin of 240 wine samples from four different production regions in France within the regional and sub regional ranges^5^. Mattia Rapa et al. used ICP-MS technology to conduct multi-element screening analysis on soil and wine samples. They selected 10 elements, including Ni and Cs, from 45 elements as the main information variables for origin tracing. Combined with chemometric classification methods, they were able to accurately classify Piemonte and Sicily samples^6^.

However, in terms of large-scale origin testing of wine samples, the aforementioned methods encounter challenges such as labor-intensive sample preparation, expensive laboratory equipment, and specific experimental environment requirements. Spectral technology has been widely applied in wine origin identification, wine quality evaluation^7^, and various food research^8,9^ due to its simplicity, high sensitivity, no need for sample pretreatment, and no need for experimental reagents. Lu et al. identified corresponding biomarkers by searching for Raman spectra of red wine, analyzed Raman spectra using PCA, and established a red wine origin recognition model by combining dimensionality reduction data with deep learning, achieving a more accurate classification of red wine origins^10^. Tana et al. used ultra-high performance liquid chromatography quadrupole time-of-flight mass spectrometry to identify characteristic substances in wine from different regions, screened out the characteristic bands of near-infrared spectroscopy of wine, and accurately divided wine samples from six regions^11^. Daniel et al. established a discriminative model for 64 white wine samples from Australia and New Zealand by spectral method, with an accuracy of 86%^12^.

Although the above method can quickly and conveniently achieve origin traceability by utilizing the spectral signal of wine, when using traditional spectral analysis methods to analyze spectral signals, there is a phenomenon of aliasing of signals from different components, making it difficult to extract useful information from the spectrum^13,14^. The proposal of two-dimensional correlation spectrum effectively makes up for the problem of poor resolution of traditional one-dimensional Spectral resolution ^15,16^. In addition, because deep learning can improve the performance and accuracy of the model by automatically identifying the correlation of the original data without human intervention^17^, in other fields, deep learning is often used as an auxiliary method for the research of two-dimensional correlation spectral images. For example, Dong et al. combined the two-dimensional correlation spectrum of Lycium barbarum near-infrared hyperspectral image with convolutional neural network to establish the origin traceability model of Lycium barbarum origin, providing key technical support for the development of Lycium barbarum industry^18^. Liu et al. used two-dimensional correlation spectral images combined with residual convolutional neural network to effectively classify different origins and parts of Panax notoginseng, providing a feasible strategy for quality control of traditional chinese medicine^19^.

Using near-infrared two-dimensional correlation spectral images for wine origin traceability research can enhance spectral resolution. However, the large amount of irrelevant signals contained in the binary correlation spectra generated from the original spectral data can seriously affect detection accuracy. To address this issue, a method is proposed to screen spectral signal characteristic bands using metabolomics. Firstly, the main differential substances in wine samples from different regions are selected based on UPLC-Q-TOF-MS experimental results. Then, the characteristic bands of the wine's near-infrared spectrum are extracted. Finally, a generalized two-dimensional correlation spectral image analysis is performed on the two-dimensional correlation spectrum to establish a CNN origin classification model and achieve fast traceability detection of wine origin.

**Figure 1:**
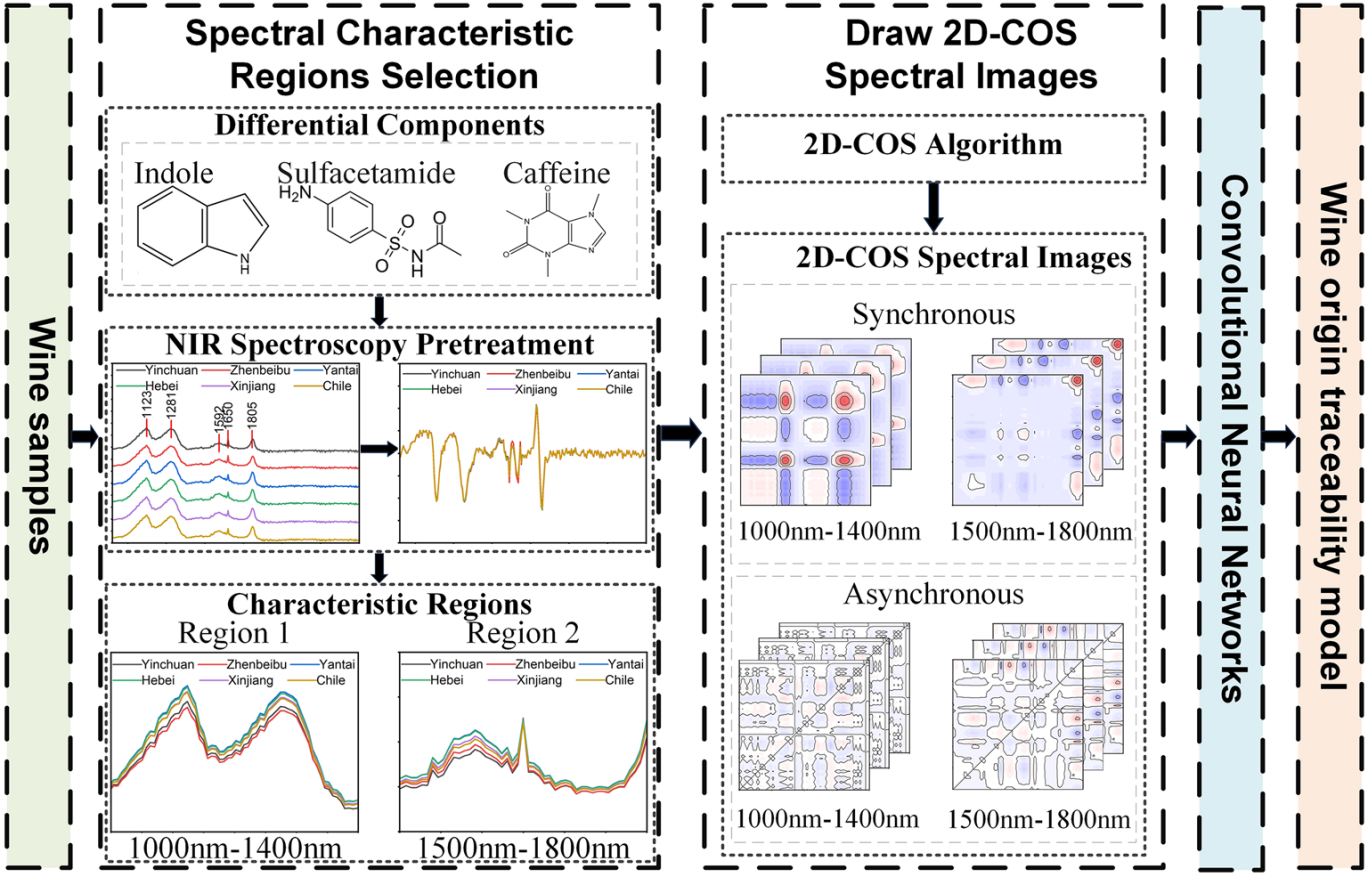
Technology roadmap.

The Technology roadmap of this article is shown in Fig. 1.

## Materials and methods

### Wine sample selection and near-infrared spectroscopy collection

The samples used in the experiment were from six different regions, namely Yinchuan and Zhenbeibu in Ningxia, Changli in Hebei, Yantai in Shandong, Turpan in Xinjiang, and Limari Valley in Chile. 30 samples were collected from each production area, totaling 180 samples. In order to ensure the reliability of the experimental results, the wine samples used in the experiment were all finished wines made from Cabernet Sauvignon grapes through temperature controlled fermentation and aging in oak barrels.

The NIR test platform is composed of NIR2500 (Ideaoptics Instruments Co., Ltd., China), HL2000-12 halogen light source, RIB-600-NIR direct optical fiber,R4 color dish spectral measuring stand and Morpho software(Version 3.2 12.2, Available from http://www.ideaoptics.com).

The experiment is carried out in a constant temperature and humidity environment. The wine samples are opened after 10 min’rest, placed in quartz cell, and the wine samples are collected using a near-infrared spectroscope. The wavelength range of the near-infrared spectrum collected by the wine samples ranged from 900 to 2500 nm, the wavelength resolution is 3.2 nm, the integration time is 1 ms–120 s, and the signal-to-noise ratio is 7500:1. The collection time for each spectral scan is 10 s. The total collection time for near-infrared spectroscopy of all samples is 50 min.

### UPLC-Q-TOF-MS experimental method

The Q-TOF equipment used in the experiment is Agilent High Resolution Liquid Mass Spectrometry (HRLC-MS) system (Agilent Technologies, Santa Clara, CA, United States). The main components of wine are extracted and analyzed by MassHunter B.06.00 (Agilent Technologies, Inc. 2006–2019, Santa Clara, CA, United States) and Mass Profiler Professional 12.5 software (Agilent Technologies, Santa Clara, CA, United States) (Version 12.5, Available from www.agilent.com.cn) to extract and analyze 130 main components of wine. Experimental reagents include distilled water, Ammonium formate (chromatographically pure) and methanol (chromatographically pure).

The experimental methods are as follows: Firstly, 1 mL sample is accurately measured in a 1.5 mL centrifugal tube, centrifuged at 4 °C for 10 min at 10,000 rpm, and passed through 0.22 μM Microporous filter, on-machine detection. The chromatographic column is Agilent Eclipse Plus C18 (3 × 150 mm, 1.8 μM). Column temperature: 40 °C; Automatic sampler temperature: 4 °C; Input: 2μL; Flow rate: 0.3 mL/min; Column balance time: 0.5 mL/min; Analysis time: 20 min. The mobile phase is 5 mmol/L ammonium formate aqueous phase and methanol phase.

In each data collection cycle, parent ions with an intensity greater than 5000 are screened. TOF-MS scan time is 150 ms, quality detection range is 50–1000 Da, collected in HighSensitivity mode.

### Spectral data preprocessing

In addition to the feature data of the detected samples, the original near-infrared spectral data also contains many redundant variables and noise signals caused by external interference^20^. In order to eliminate interference and establish a highly reliable two-dimensional correlated infrared spectrum, MSC + S-G + FD was used to preprocess the original spectrum in the experiment to deduct the impact of instrument background or drift on the spectral signal, eliminate spectral differences caused by scattering effects caused by uneven particle sizes in the wine liquid during the spectral data collection process^21^, and reduce spectral signal noise and improve spectral signal to noise ratio.

**Figure 2:**
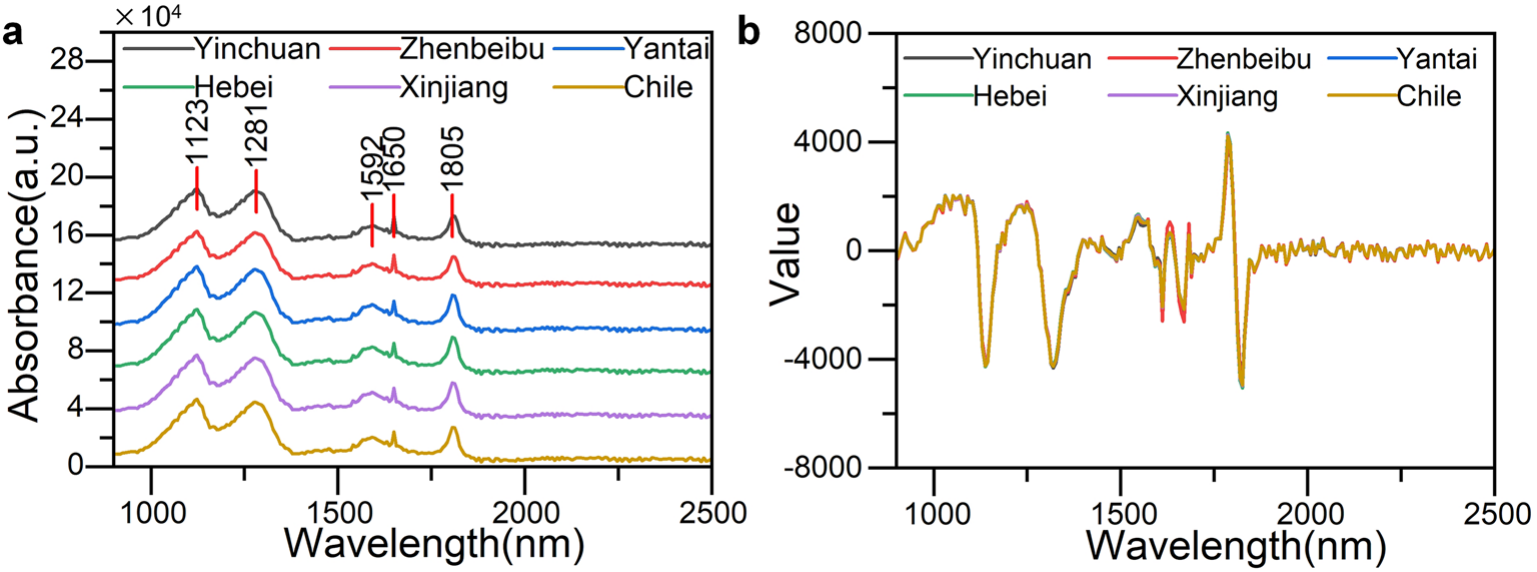
(**a**) Original near-infrared spectra from six production areas, (**b)** average near-infrared spectra after MSC + S-G + FD pretreatment.

Figure 2a shows the original average near-infrared spectra of six wine samples from different regions in the range of 900–2500 nm. Except for five peaks at 1123 nm, 1281 nm, 1592 nm, 1650 nm, and 1805 nm with slight differences in absorbance values, the other bands are basically similar. Figure 2b shows the average near-infrared spectra of 6 production areas after MSC + S-G + FD pretreatment.

### 2D-COS image acquisition

The acquisition of the generalized two-dimensional correlation infrared spectrum refers to the recording of the corresponding infrared spectrum (i.e. dynamic spectrum) of the sample under the disturbance state and the correlation analysis of a series of dynamic spectra when external disturbances (such as electrical, magnetic, thermal, mechanical, chemical, concentration and composition changes) are applied to the sample to be tested, and then the results are presented in a two-dimensional Contour line map or three-dimensional figure,the process of obtaining two-dimensional correlated infrared spectral images^17^.

Strictly speaking, dynamic spectrum refers to the result of subtracting a reference spectrum from the spectrum of a sample in different states due to interference. The dynamic spectrum with disturbances (electrical, magnetic, thermal, mechanical, chemical, concentration, and composition changes, etc.) can be recorded as Eq. (1):

1 $$y(v,t) = \left\{ \begin{gathered} x(v,t) - \overline{x}(v),T_{\min } \le t \le T_{\max } \hfill \\ 0,otherwise \hfill \\ \end{gathered} \right.$$

$x(v,t)$ represents the spectral intensity of the sample at variable $v$ under disturbance $t$, while $\overline{x}(v)$ represents the reference value of spectral intensity at variable $v$. In general, the average spectral intensity value at variable $v$ in the sample spectrum obtained from the entire disturbance process (from $t$ = $T_{\min }$ to $t$ = $T_{\max }$) can be taken as $\overline{x}(v)$. Equation (2):

2 $$\overline{x}(v) = \frac{1}{{(T_{\max } - T_{\min } )}}\int_{{T_{\min } }}^{{T_{\max } }} {x(v,t)} dt$$

or a specific value can be selected as $\overline{x}(v)$, such as the spectral intensity value at variable $v$ in the sample spectrum when $t$ = $T_{\min }$ or $t$ = $T_{\max }$. At this point, the reference point is the initial or end state of the experiment. When the reference point is simply set to 0, the dynamic spectrum is the spectral intensity observed at the current moment.

Before calculating the generalized two-dimensional correlation spectrum, it is necessary to perform a Fourier transform on the dynamic spectrum $y(v,t)$, and the calculation of the generalized two-dimensional correlation spectrum obtained from cross correlation analysis is shown in Eq. (3):

3 $$Y_{1} (\omega ) = \int_{ - \infty }^{ + \infty } {y(v_{1} ,t)} e^{ - i\omega t} dt$$

4 $$\Phi (v_{1} ,v_{2} ) + i\Psi (v_{1} ,v_{2} ) = \frac{1}{{\pi (T_{\max } - T_{\min } )}}\int_{0}^{\infty } {Y_{1} (\omega )} Y_{2}^{*} (\omega )d\omega$$

The real part $\Phi (v_{1} ,v_{2} )$ in Eq. (4) is called synchronous correlation spectrum, and the imaginary part $\Psi (v_{1} ,v_{2} )$ is called asynchronous correlation spectrum.

The synchronous correlation spectrum represents the similarity change in spectral intensity between two variables as a function of disturbance. Asynchronous correlation spectra represent changes in spectral intensity with disturbance, differences in spectral intensity between two variables, or phase differences in spectral intensity between two variables.

In practical experiments, it is necessary to transform the integration formula under finite and discrete experimental values. Assuming that m data points are measured at equal intervals under disturbance $t$, the synchronous correlation spectrum at this time can be expressed as Eq. (5):

5 $$\Phi (v_{1} ,v_{2} ) = \frac{1}{m - 1}\sum\limits_{j = 1}^{m} {y(v_{1} ,t_{j} )} \cdot y(v_{2} ,t_{j} )$$

The calculation of asynchronous correlation spectra can be expressed as Eq. (6):

6 $$\Psi (v_{1} ,v_{2} ) = \frac{1}{m - 1}\sum\limits_{j = 1}^{m} {y(v_{1} ,t_{j} )} \cdot \sum\limits_{j = 1}^{m} {M \cdot y(v_{2} ,t_{j} )}$$

The $M$ in Eq. (6) represents an n-th order Hilbert Noda matrix,and its expression is:

7 $$M_{jk} = \left\{ {\left. \begin{gathered} 0,j = k \hfill \\ \frac{1}{\pi (k - j)},j \ne k \hfill \\ \end{gathered} \right\}} \right.$$

In the process of obtaining synchronous and asynchronous correlation spectral images of samples in the experiment, the average spectrum of the samples is first obtained, and then the characteristic band regions of the spectra are selected based on the Q-TOF results. The spectral data of each sample's characteristic region is compared with the average spectrum, and synchronous and asynchronous correlation spectral images of all samples are generated using time as the disturbance variable.

### Establishing a deep learning model

**Figure 3:**
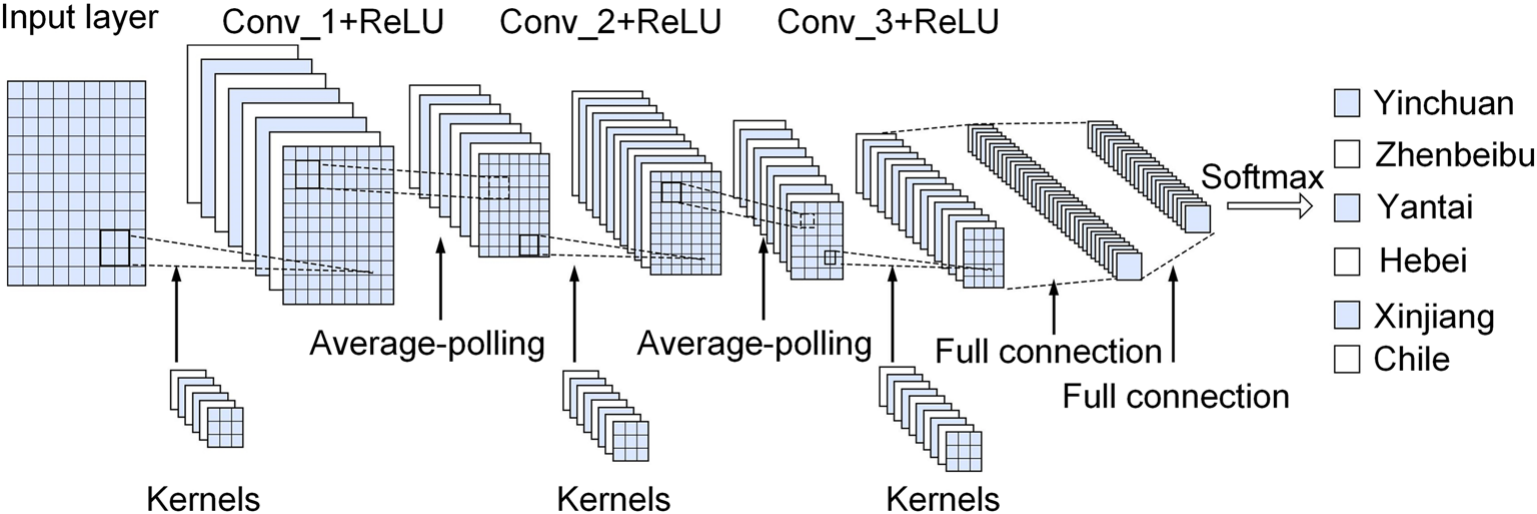
Structure diagram of Convolutional neural network model.

The deep learning model used in the experiment is Convolutional Neural Networks (CNN). CNN, as a feedforward neural network with convolutional structure, extracts the features of input data through convolutional operations. As shown in Fig. 3, the basic structure consists of an input layer, convolutional layer, pooling layer, fully connected layer, and output layer, with the structural characteristics of local area connection, weight sharing, and downsampling. Weight sharing and local area connectivity reduce the complexity of the network model and reduce the number of weights. The convolution calculation formula is:

8 $$X_{n}^{m} = f\sum\nolimits_{{i\hat{I}}} {(X_{n}^{m - 1} K_{in}^{m} + b_{n}^{m} )}$$

$m$ is the number of convolution layers and $f()$ is the Activation function. Through calculation, the $n$ feature map of layer $m$ can be obtained, $K$ represents the convolution kernel, and $\hat{I}$ represents the input image set, representing the offset matrix corresponding to the $n$ feature map of layer $m$.

**Figure 4:**
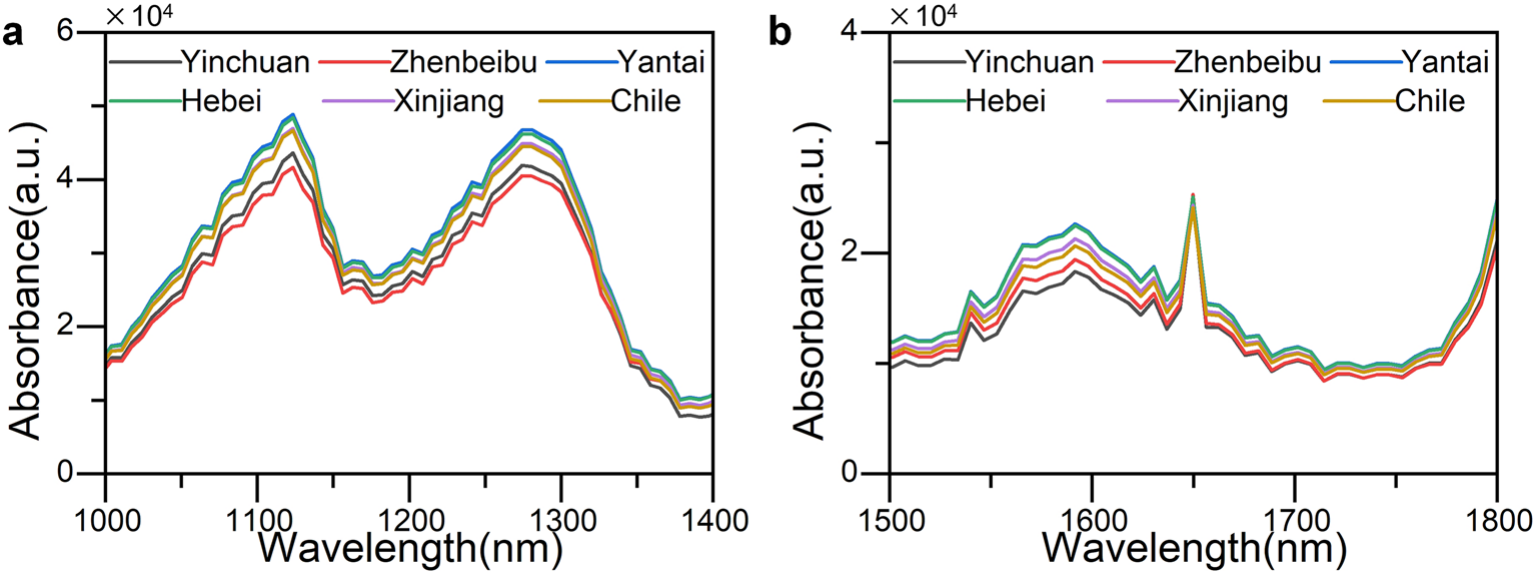
Characteristic regions (**a** 1000–1400 nm, **b** 1500–1800 nm).

The structure of the CNN model is shown in Fig. 4, the pooling layer in the structure can effectively reduce the size of the parameter matrix, improve the calculation speed and the robustness of the feature data. The activation function operates the output of the convolution layer nonlinearly to extract feature information. In this experiment, the linear rectification function (RELU) is selected as the activation function. Compared with other activation function, the sparsity of the linear rectification function can accelerate learning and simplify the model. After applying the linear rectification layer, the pooling layer performs parameter reduction to combine specific features of the convolutional layer, thereby avoiding overfitting and ensuring a stable convolutional process.

The experiment uses MATLAB 2020a to establish a CNN prediction model, uses the filtered near-infrared characteristic wavelength to construct a generalized 2D-COS image as the input, and the output layer has a neuron for regression of classification results.

## Results and discussion

### UPLC-Q-TOF-MS result analysis

According to the Q-TOF experimental method described in Section “UPLC-Q-TOF-MS experimental method”, chemical composition analysis was conducted on wine samples from six production regions. The experimental results indicate that indole, sulfacetamide, and caffeine can be used as characteristic substances for tracing the origin of experimental samples. Three substances were t-tested using SPSS software (Version 19.0, Available from https://www.ibm.com/cn-zh/spss), with p-values of 0.03, 0.02, and 0.05, respectively. The results indicate that all three substances can be used as characteristic components of the experimental wine sample. The main differential metabolites analysis results are shown in Table 1.

**Table 1:** Spectral peak area of different substances in wine from different regions.

Compounds	Yinchuan	Zhenbeibu	Hebei	Xinjiang	Yantai	Chile
Indole^22^	87.54	87.66	73.66	80.90	84.16	86.66
Sulfacetamide	72.58	75.28	82.17	88.97	82.32	73.91
Caffeine^3^	92.34	91.49	92.21	94.61	93.46	95.13

To screen the characteristic wavelength range, the structural formulas of the three characteristic substances obtained from the experiment were analyzed. Indole is an aromatic heterocyclic organic compound, consisting of a six-membered benzene ring fused to a five-membered nitrogen-containing pyrrole ring ^22,23^. Therefore, according to the IR functional group correlation table, this substance may exist within the range of 1000–1400 nm. Based on the analysis of the IR main functional group correlation table, it is known that acetamide may exist in the 1000–1400 nm range ^24^. Caffeine is a biologically active alkaloid compound of methylxanthine, with a spectral range of 1500–1800 nm.

Therefore, the spectral range of 1000–1400 nm and 1500–1800 nm are the characteristic regions in the near-infrared spectrum. The near-infrared spectra of two characteristic bands are shown in Fig. 4.

### The 2D-COS spectra images analysis

**Figure 5:**
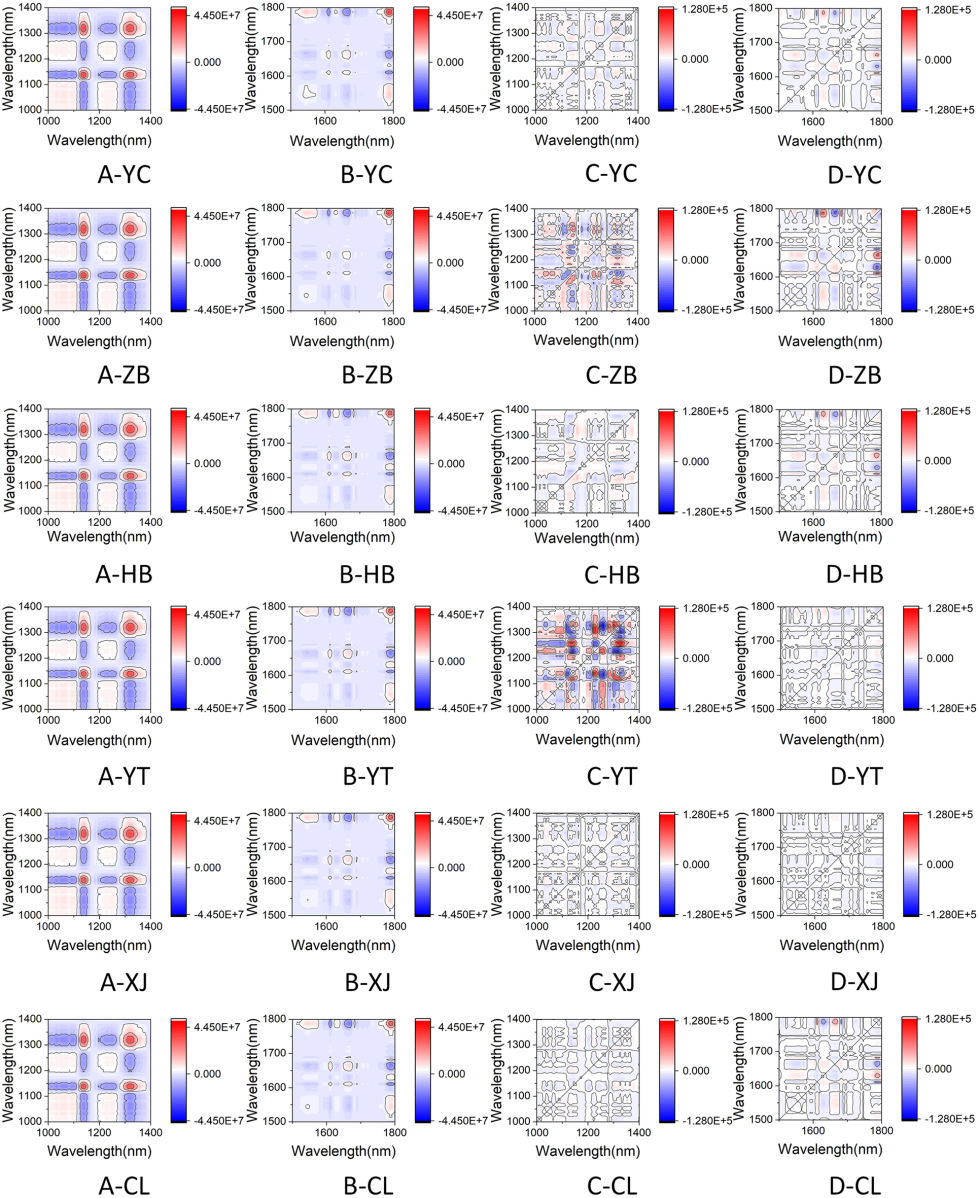
Six sources of two-dimensional correlation spectral images (**A**: synchronous correlation spectrum in the 1000–1400 nm wavelength range; **B**: synchronous correlation spectrum in the 1500–1800 nm wavelength range; **C**: asynchronous correlation spectrum in the 1000–1400 nm wavelength range; **D**: asynchronous correlation spectrum in the 1500–1800 nm wavelength range. YC: Yinchuan, Ningxia; ZB: Zhenbeibu, Ningxia; HB: Changli, Hebei; YT: Yantai, Shandong; XJ: Turpan, Xinjiang; CL: Limari Valley, Chile).

Based on the Q-TOF experimental results, 720 two-dimensional correlation spectral images were drawn in two feature band ranges, with 180 synchronous correlation spectral images and 180 asynchronous correlation spectral images for each feature band region. The synchronous and asynchronous correlation spectral images of wine from different regions in the feature regions of 1000–1400 nm and 1500–1800 nm are shown in Fig. 5A–D represent synchronous correlation spectra in the 1000–1400 nm wavelength range, synchronous correlation spectra in the 1500–1800 nm wavelength range, asynchronous correlation spectra in the 1000–1400 nm wavelength range, and asynchronous correlation spectra in the 1500–1800 nm wavelength range, respectively. YC, ZB, HB, YT, XJ, and CL represent regions of origin in Yinchuan, Zhenbeibu, Hebei, Yantai, Xinjiang, and Chile.

For synchronous correlation spectra, the peak on the diagonal is called an automatic peak, which represents the similarity of spectral intensity changes with disturbance at a certain variable ^25,26^. As shown in column 5A of Fig. 5A, there are two obvious strong automatic peaks on the diagonal of the 1000–1400 nm synchronous correlation spectrum, located at 1139 nm and 1323 nm respectively. As shown in column 5B of Fig. 5B, there is a clear strong automatic peak and multiple weak automatic peaks on the diagonal of the 1500–1800 nm synchronous correlation spectrum, with the strong automatic peak located at 1788 nm. It indicates that the spectral intensity changes of the samples at 1139 nm, 1323 nm, and 1788 nm are consistent, with the maximum similarity change, and the automatic peak has the maximum positive value. The peaks on the non diagonal lines of the synchronous correlation spectrum are called cross peaks, and their intensity can be positive or negative, indicating the positive or negative correlation of spectral intensity changes at the variable under disturbance. As shown in column 5A of Fig. 5A, there is a positive strong cross peak at 1138 nm and 1320 nm, indicating a positive correlation in the spectral intensity change here. There are negative strong cross peaks at 1233 nm and 1320 nm, 1248 nm and 1320 nm, 1319 nm and 1090 nm, indicating a negative correlation change in spectral intensity.

The asynchronous correlation spectrum represents the difference in spectral intensity changes between two variables. There are no automatic peaks in the asynchronous correlation spectrum, and only cross peaks appear when the intensities of the two dynamic spectra undergo non phase (delayed or accelerated) changes. Generally speaking, asynchronous correlation spectra have higher resolution than synchronous correlation spectra, but due to complex cross peaks, the asynchronous correlation spectra of some complex mixtures are often difficult to interpret. From columns C and D, it is not difficult to see that wine samples from different regions within the same wavelength band have significant differences in asynchronous correlation spectra, making it difficult to accurately distinguish them based on vision. Therefore, it is necessary to further analyze using convolutional neural network models.

### Analysis of CNN model results

Randomly divide the synchronous correlation spectral images and asynchronous correlation spectral images of each feature band from each production area into 2/3 training sets and 1/3 testing sets. Each time the dataset was randomly divided, out of a total of 720 two-dimensional spectral images of various types in each characteristic band of the six production areas, 480 were used as the training set and 240 were used as the test set. To avoid the randomness of single modeling results, the samples were randomly divided into 50 training and testing sets, and CNN origin traceability models for different varieties of wine were established. The average classification accuracy of the 50 sets was taken as the model accuracy.

To demonstrate that the classification performance of the model established by combining two-dimensional correlated spectral images with CNN is superior to that established by using one-dimensional near-infrared spectroscopy and traditional classification methods, two feature wavelengths were combined with LDA^27–29^ algorithm and SVM^30,31^ algorithm to establish a classification model. The near-infrared spectra of each feature band were still randomly divided into 2/3 training sets and 1/3 test sets 50 times for modeling, and take the average of 50 classification accuracies as the model accuracy.

The experimental results are shown in Table 2. The accuracy of the CNN model test set established using asynchronous correlation spectral images drawn in two characteristic bands of 1000–1400 nm and 1500–1800 nm is 0.96 and 0.93, respectively. The results are superior to the LDA model and SVM model's 0.91, 0.83, 0.90 and 0.86, and their classification accuracy is basically consistent with the training set, indicating that the established model is stable and reliable. The CNN model established for synchronous correlation spectral images of wine has test set accuracy of 0.91 and 0.87 in the two feature bands of 1000–1400 nm and 1500–1800 nm. Compared to the classification model established for asynchronous correlation spectral images in the same feature band, the accuracy of the classification model is lower, but the classification results are still better than traditional LDA and SVM models.

**Table 2:** LDA, SVM, CNN model classification results.

		LDA	SVM	Synchronous + CNN	Asynchronous + CNN
		Accuracy	Accuracy	Accuracy	Accuracy
Train set	1000–1400 nm	0.91	0.92	0.92	0.96
	1500–1800 nm	0.89	0.91	0.89	0.95
Test set	1000–1400 nm	0.91	0.90	0.91	0.96
	1500–1800 nm	0.83	0.86	0.87	0.93

The results indicate that the model established using asynchronous correlation spectral images has the strongest generalization ability and the highest classification accuracy, making it more suitable for establishing deep learning models based on image processing to identify different regions of wine. Due to the higher resolution of the preprocessed asynchronous correlation spectral image compared to the synchronous correlation spectrum, and the relatively complex non phase changes occurring in the cross peaks, the spectral resolution is more pronounced, and there are significant differences between samples of the same type, which is more conducive to distinguishing CNN models. The synchronous correlation spectrum essentially provides the same information as the one-dimensional spectrum. Although it can more clearly show the small peaks that were previously mixed in the one-dimensional spectrum when extended to two-dimensional, it has high consistency in phase changes, making the synchronous correlation spectrum images of different regions extremely similar and difficult to effectively distinguish. In addition, the accuracy of the three models based on the 1000–1400 nm feature region is higher than that based on the 1500–1800 nm feature region. This may be because the 1000–1400 nm feature band is determined by two different substances, and the number and types of molecular functional groups are more than the 1500–1800 nm feature band, providing more feature components in the infrared spectrum.

Summarizing the above experimental results, the synchronous correlation spectral images drawn based on two feature bands are more suitable for establishing deep learning models, which contain more information suitable for wine traceability. At the same time, the synchronous correlation spectral image drawn in the 1000–1400 nm feature area has higher accuracy, which can more effectively distinguish wine samples from different regions, thereby simplifying the model complexity. This method can effectively trace wine from different origins in cases of significant sample differences, providing a possible method for protecting wine geographical indications and improving the wine traceability system.

## Conclusions

This experiment combines metabolomics analysis methods with near-infrared spectroscopy to locate the differential substances in wine from different regions based on UPLC-Q-TOF-MS analysis results. By analyzing the main chemical bonds of the differential substances, the corresponding spectra of the main infrared functional groups are searched, and the characteristic regions of near-infrared spectroscopy are screened. While effectively retaining more useful spectral information, irrelevant variables in the spectrum are removed as much as possible. In addition, the two-dimensional correlation spectral images drawn from the selected feature bands were combined with CNN to transform the complex one-dimensional spectral data analysis process into a relatively simple two-dimensional correlation spectral image processing problem, which was superior to traditional spectroscopy research and effectively avoided the common wave peak aliasing problem in near-infrared spectroscopy. For the collected wine samples from six different regions, this method has significant advantages in wine origin traceability and has high stability, providing a new method for wine origin traceability.

## Data Availability

The datasets generated and/or analyzed during the current study are not publicly available due to further investigation running on the same project for futuristic solutions but are available from the corresponding author on reasonable request.
